# 25-Hydroxycholesterol protecting from cerebral ischemia-reperfusion injury through the inhibition of STING activity

**DOI:** 10.18632/aging.203337

**Published:** 2021-08-18

**Authors:** Feihong Lin, Xinyu Yao, Chang Kong, Xia Liu, Zhangfan Zhao, Suhuan Rao, Lu Wang, Shan Li, Junlu Wang, Qinxue Dai

**Affiliations:** 1 Department of Anesthesiology, The First Affiliated Hospital of Wenzhou Medical University, Wenzhou, Zhejiang, China

**Keywords:** middle cerebral artery occlusion, 25-hydroxycholesterol, STING, autophagy, mTOR

## Abstract

Middle cerebral artery occlusion (MCAO) injury refers to impaired blood supply to the brain that is caused by a cerebrovascular disease, resulting in local brain tissue ischemia, hypoxic necrosis, and rapid neurological impairment. Nevertheless, the mechanisms involved are unclear, and pharmacological interventions are lacking. 25-Hydroxycholesterol (25-HC) was reported to be involved in cholesterol and lipid metabolism as an oxysterol molecule. This study aimed to determine whether 25-HC exerts a cerebral protective effect on MCAO injury and investigate its potential mechanism. 25-HC was administered prior to reperfusion in a mouse model of MCAO injury. 25-HC evidently decreased infarct size induced by MCAO and enhanced brain function. It reduced stimulator of interferon gene (STING) activity and regulated mTOR to inhibit autophagy and induce cerebral ischemia tolerance. Thus, 25-HC improved MCAO injury through the STING channel. As indicated in this preliminary study, 25-HC improved MCAO injury by inhibiting STING activity and autophagy as well as by reducing brain nerve cell apoptosis. Thus, it is a potential treatment drug for brain injury.

## INTRODUCTION

Ischemic stroke is a general disease associated with the neural mechanism. It has high mortality and disability rates. This disease causes physical disability and serious cognitive damage, often requiring immediate treatment. Moreover, patients undergoing cerebrovascular surgery (e.g., intervention and aneurysm clipping) are at risk of cerebral ischemia due to vasospasm [1]. If cerebral ischemia occurs, the perfusion process is significantly reduced, and the cell starts to swell and die. Perfusion is required to replace restricted time window to avoid deterioration of neurological functions [2]. Existing clinical interventions are subject to limitations in the time window for treatment. Therefore, the research and development of new treatments for stroke are significant challenges for scientists.

25-Hydroxycholesterol (25-HC) is one of the oxysterols (oxidized forms of cholesterol). Oxysterols participate in cholesterol homeostasis, immune response, and pathogenesis of several diseases such as neurodegenerative diseases and atherosclerosis [3]. The brain is the most cholesterol-rich organ in the body and contains approximately 25% of the total amounts [4, 5]. The major portion of this cholesterol is present in myelin, a lamellar arrangement of oligodendrocyte plasma membranes that acts as an electric insulator along the length of large axons. Accumulating epidemiological and molecular evidence indicates a clear link between cholesterol turnover and neurodegenerative diseases– hypercholesterolemia per se is an important risk factor for Alzheimer’s disease and Parkinson’s disease [6]. In a study on patients with mild cognitive impairment, the level of 24S-OHC appeared to be the most sensitive marker [7, 8]. Moreover, studies have shown that 25-HC could protect against reperfusion injury-induced myocardial apoptosis by inhibiting the poly (ADP-ribose) polymerase activity [9]. However, the role of 25-HC in middle cerebral artery occlusion (MCAO) injury remains unclear.

Autophagy is a basic cellular degradation system involved in the establishment of homeostasis. It is considered a distinct form of programmed cell death that differs from apoptosis [10]. Autophagy is an induced response in terms of stress, including inflammation, exposure to cigarette smoke, and hypoxia. Dysfunctionality-based and senescent cell organs or cytoplasmic components are encapsulated through autophagosomes and then transferred to lysosomes for processing [11]. However, when autophagy flux is disrupted, the accumulation of damaged proteins or cell organs (e.g., mitochondria) destroys the lung tissues. Complete autophagy is dependent on common lysosomal functions, and the inhibition of autophagosomal degradation attributed to lysosomal damage may lead to autophagy dysfunction [12]. Autophagy, as a degrading/recycling mechanism, is considered to critically affect the pathologically related conditions of numerous organs (e.g., cerebral ischemia). Stimulator of interferon genes (STING) is an important signaling molecule in immunity and inflammation and acts as a vital regulator of autophagy [13]. Abdullah reported that after traumatic brain injury, the expression of STING is upregulated in the cortex and striatum of wild-type mice and mediates neuroinflammatory response. Meanwhile, neuroinflammation in the brain tissue of STING gene knockout mice is mitigated, and the nerve injury is significantly improved. This suggests that STING-activated autophagy is involved in cerebral ischemia-reperfusion (IR) injury.

With the above background, this study aimed to elucidate the role of 25-HC in brain MCAO injury and the probable mechanisms involved in this process.

## RESULTS

### 25-HC reduced brain infarction in the MCAO model mice

**Figure 1:**
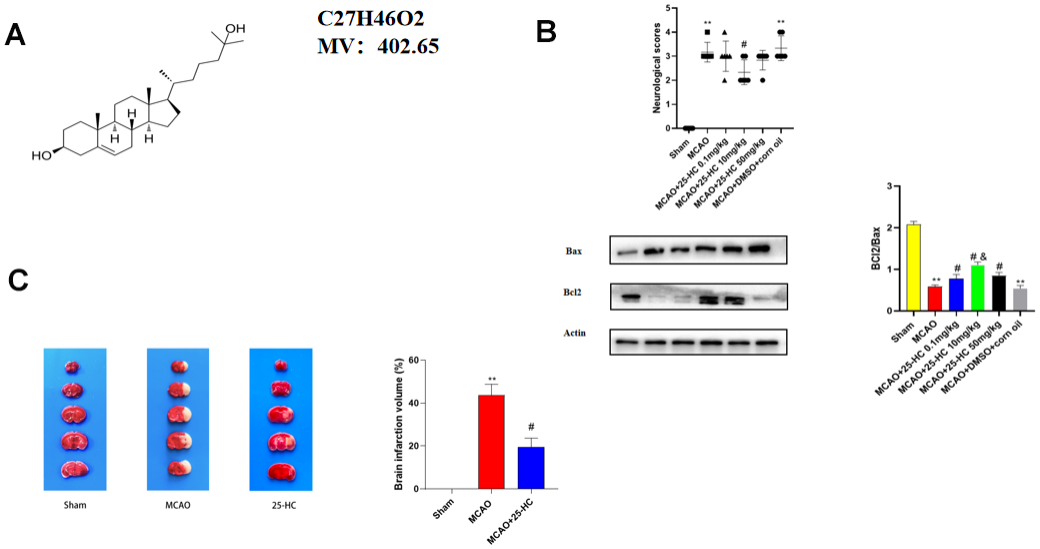
**25-Hydroxysterol reduces size of cerebral infarction in a mouse middle cerebral artery occlusion (MCAO) model.** Mice were randomly divided into three groups. Control group, healthy mice; MCAO group, mice with MCAO; mice with MCAO treated with + 25-Hydroxymethosterol. (**A**) Chemical structure of 25-Hydroxysterol. (**B**) Neurobehavioral score and Western blot analysis of BCL2 and Bax expression to select drug concentration (**P < 0.01 versus Sham group, #P <0.05 versus MCAO group, &P<0.05 versus MCAO+25-HC 0.1mg/kg group). (**C**) TTC staining was used to detect cerebral infarction in mice (N = 5 per group, **P<0.001 versus sham group, #*P*<0.05 versus MCAO group).

This study employed TTC staining to estimate the severity of cerebral infarction. Specific to stained sections, the common tissue appeared to be dark red, and the infarct area turned white. Figure 1A shows the chemical structure of 25-HC. As indicated in Figure 1B, Compared with other drug concentrations, 10mg/kg drug concentration can better improve the neurobehavioral score and increase the ratio of Bax/Bcl2, so we chose this drug concentration for the subsequent experiment. As presented in Figure 1C, cerebral infarction volume remarkably increased in the MCAO group than in the sham group (*P*<0.01). Nevertheless, after treatment with 25-HC, the infarct volume decreased noticeably (*P*<0.05).

### 25-HC inhibited MCAO-induced apoptosis of brain tissues

**Figure 2:**
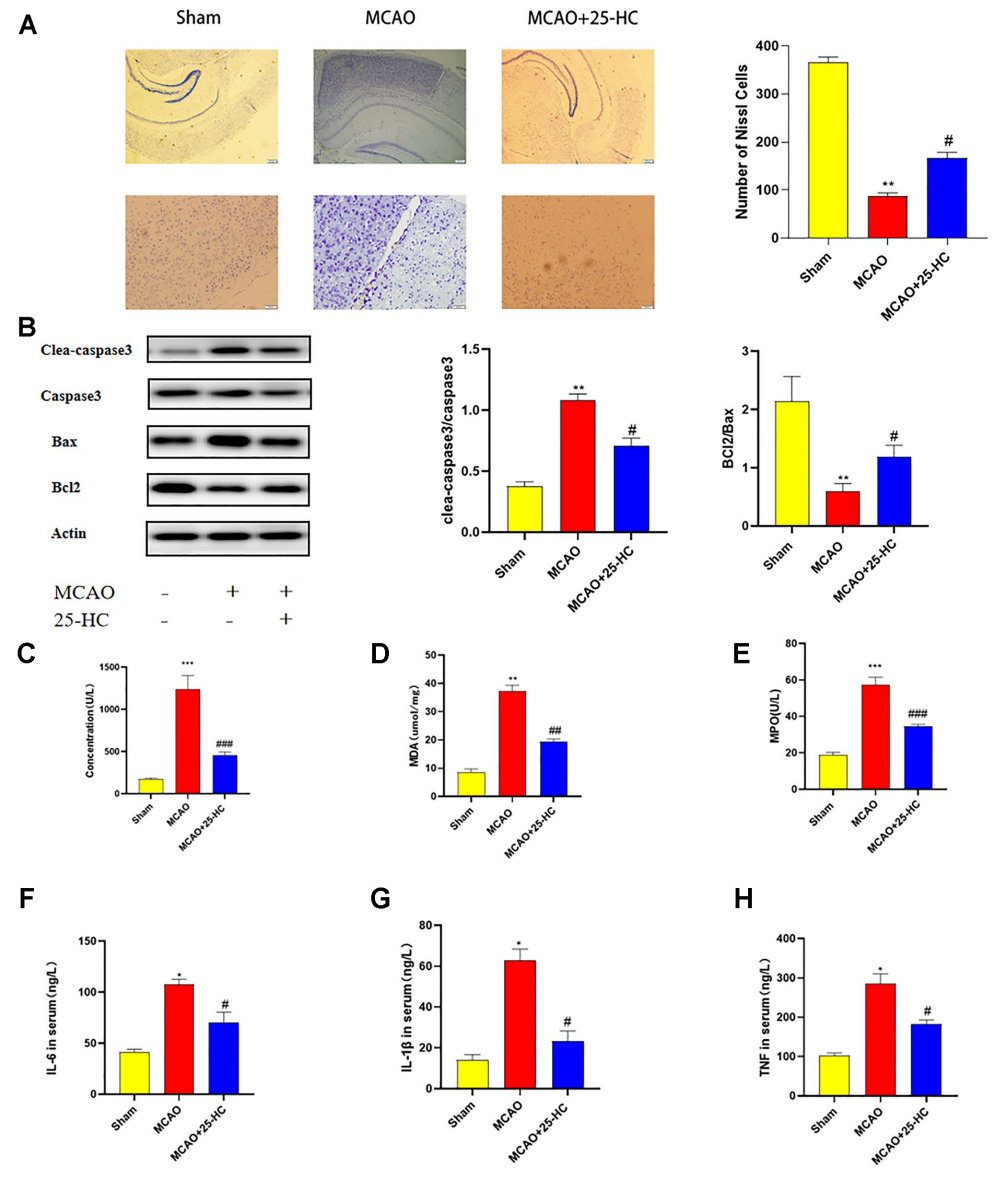
**25-Hydroxysterol reduces brain tissue apoptosis and oxidative damage and inflammation in mouse middle cerebral artery occlusion (MCAO) model.** (**A**) Nissl staining 40× and 200× (scale bar = 50 μm/200 μm, N = 4 per group). (**B**) Western blots were used to detect the expressions of caspase-3, cleaved caspase-3, Bcl2, and Bax. (**C**–**H**) Serum levels of interleukin (IL-6), IL-1β, and tumor necrosis factor-α were detected by commercially available ELISA kits. Myeloperoxidase (MPO), malondialdehyde (MDA), and lactate dehydrogenase (LDH) levels in the serum were quantified by using commercial kits following instructions of the manufacturer. (N = 6 per group, **P<0.01 versus sham group, ##*P*<0.01, #*P*<0.05 versus MCAO group).

To investigate the influence of 25-HC on the apoptosis of brain tissues in mice with MCAO, Nissl staining and Western blot were performed to detect the protein expression of caspase-3, cleaved caspase-3, Bcl-2, and Bax. As shown in Figure 2A, in the cortex, Nissl bodies in neurons of mice in the sham group were located in the cytoplasm. Pyramidal neurons were stained blue, with normal cell morphology and a compact and orderly arrangement. The injured neurons in the MCAO group exhibited atrophied neuronal cell bodies and pyridosed nuclei. In addition, 25-HC could increase the number of neurons when compared with that in the MCAO group. As indicated in Figure 2B, the protein levels of cleaved caspase-3 and Bax increased in the MCAO group, whereas those of Bcl-2 decreased; caspase-3 showed no significant change (*P*<0.01). 25-HC reversed the expression of cleaved caspase-3, Bax, and Bcl-2 in the MCAO group (*P*<0.05). Figure 2B shows that the expressions of cleaved caspase-3 and Bax was enhanced, and the expression of Bcl-2 was reduced after MCAO (*P*<0.01). In contrast to the MCAO group, 25-HC inhibited the expressions of cleaved caspase-3, Bax, and Bcl-2 (*P*<0.05). These results suggest that 25-HC can inhibit MCAO-induced apoptosis in brain tissues.

### 25-HC mitigated the inflammation and oxidative injury in mouse MCAO model

To detect changes in inflammatory factors and oxidative damage, we examined changes in the serum levels of LDH, MDA, MPO, IL-6, TNF-α, and IL-1β levels in each group. As indicated in Figure 2C–2E, LDH, MDA, and MPO activities in the MCAO group were significantly increased (*P*<0.01). 25-HC inhibited the increase in LDH, MDA, and MPO levels in the serum (*P*<0.05). In addition, the levels of IL-6, TNF-α, and IL-1β in the MCAO group were significantly increased (*P*<0.01; Figure 2E–2H). Levels of IL-6, TNF-α, and IL-1β decreased in the MCAO+25-HC group when compared with those in the MCAO group (*P*<0.05). These results suggest that 25-HC can reduce oxidative damage and inflammatory response induced by MCAO.

### 25-HC suppressed MCAO-induced enhanced autophagy

**Figure 3:**
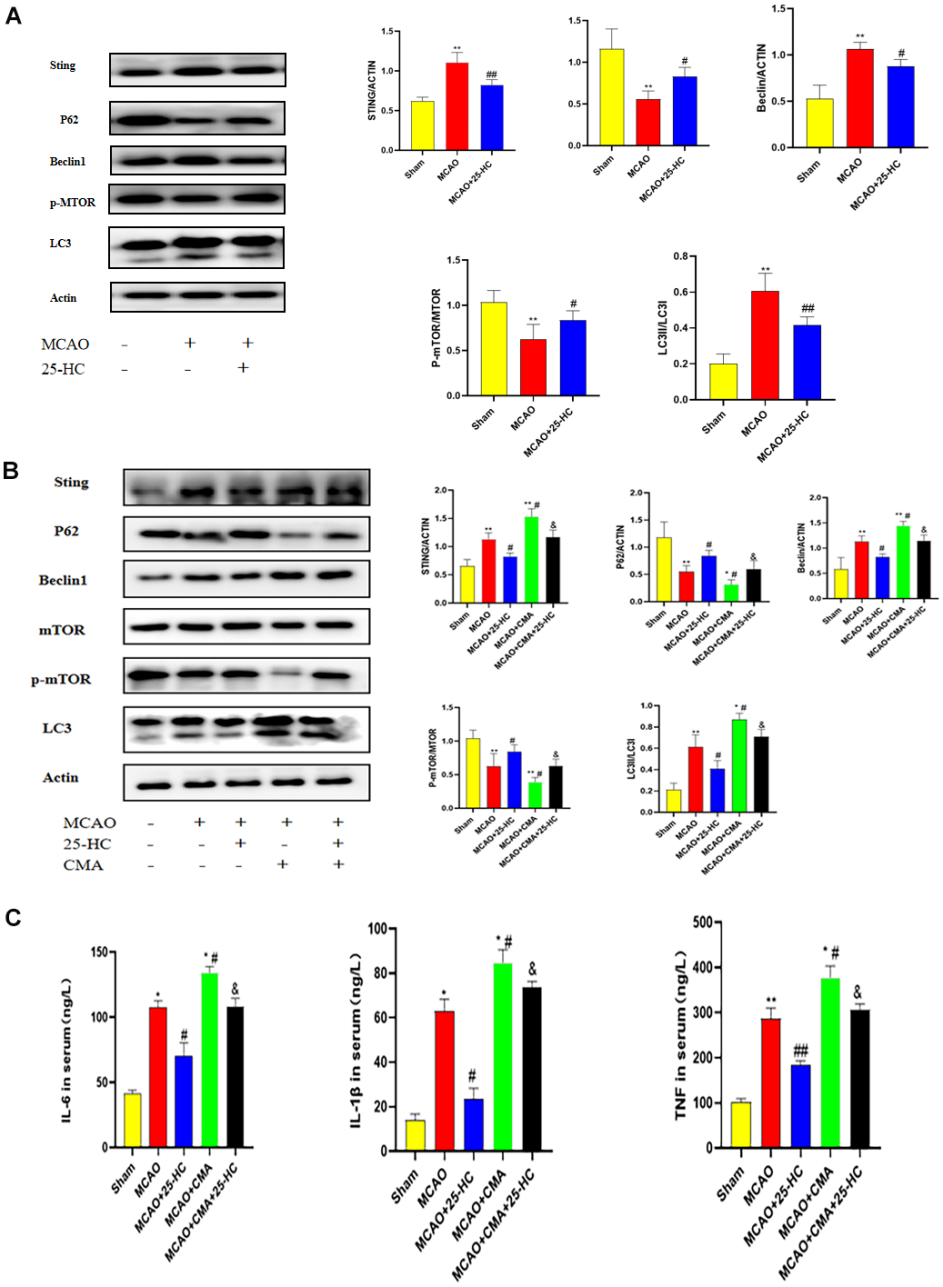
**25-Hydroxycholesterol (25-HC) suppressed the middle cerebral artery occlusion (MCAO)-induced enhanced autophagy, and the effects of 25-HC on autophagy, oxidative injury, apoptosis, and inflammation were reversed by oxygen-10-acridine acetic acid (CMA).** (**A**) Expression of p-mTOR, STING, P62, Beclin1, LC3 I, and LC3 II were tested by Western blot (N = 5 per group, **P<0.001 versus sham group, ##*P*<0.001, #*P*<0.05 versus MCAO group). (**B**) Expression of p-mTOR, STING, P62, Beclin1, LC3 I, and LC3 II were tested by Western blot (N = 5-6 per group, **P<0.01, ***P<0.001, *P<0.05 versus sham group, versus MCAO group). (**C**) Levels of interleukin (IL)-6, tumor necrosis factor-α, and IL-1β were measured by commercially available ELISA kits (N = 6 per group, **P<0.01 versus sham group, ##*P*<0.01, #*P*<0.05 versus MCAO group, *P*<0.05 versus MCAO+CMA group).

To analyze the effect of 25-HC on autophagy in MCAO mice, the expression of autophagy-related proteins was detected by Western blot. As indicated in Figure 3A, the expression of STING in the MCAO group was increased (*P*<0.01). The expression of p-mTOR was decreased after MCAO (*P*<0.01). Treatment with 25-HC inhibited the expression of STING and decreased the expression of p-mTOR (*P*<0.05). Moreover, 25-HC inhibited the increase of the expression of Beclin1 and decrease the expression of p62 in the MCAO group (*P*<0.05). In addition, 25-HC reduced the LC3 II/LC3 I ratio (*P*<0.01).

### Effects of 25-HC on autophagy, oxidative injury, apoptosis, and inflammation were reversed by CMA

CMA, an agonist of STING, was injected intraperitoneally into MCAO mice to investigate the protective mechanism of 25-HC. In contrast to the MCAO group, after CMA treatment, the expression levels of p-mTOR and p62 in the MCAO+CMA group were lower than those in the MCAO group, and STING, Beclin1, and Lc3 II/Lc3 I ratios were higher than those in the MCAO group (*P*<0.05). Moreover, in the 25-HC+CMA+MCAO group, the expressions of p-mTOR, Beclin1, p62, LC3 II, and LC3 I changed (Figure 3B, *P*<0.05). In addition, IL-6, TNF-α, and IL-1β levels in the MCAO+CMA group were significantly higher than those in the MCAO group (Figure 3C, *P*<0.05). CMA might change the effect of 25-HC on the secretion of IL-6, TNF-α, and IL-1β. In brief, as revealed in the aforementioned results, 25-HC inhibits autophagy enhancement in mice with cerebral infarction induced by MCAO through regulation of the mTOR pathway by STING.

### 25-HC protected PC12 cells from OGD/R-induced cytotoxicity

To evaluate the protective effect of different concentrations of 25-HC on PC12 cells, 25-HC (1, 10, and 100 μm) was used for cell pretreatment for 1 h, and OGD/R was the initiated. As indicated in Figure 4A, the survival rate of cells with 25-HC at 10 μM was significantly enhanced after OGD/R. In contrast to the I/R group, 25-HC (10 μM) significantly reduced the apoptosis rate. Apoptosis was detected using flow cytometry (Figure 4B–4F), and the apoptosis rate of PC12 cells in the OGD/R group was significantly higher than that in the control group. 25-HC at 10 μM was suggested to exert the most evident protective effect on cell survival rate at different drug concentrations; therefore, 10 μM was selected. To investigate the effect of 25-HC on the apoptosis of PC12 cells, the protein expression of cleaved caspase-3 was detected by immunocytochemistry staining (Figure 4G). Moreover, protein expressions of cleaved caspase-3, caspase-3, Bcl-2, and Bax were detected by Western blot. As indicated in Figure 4H, the expression of cleaved caspase-3 was enhanced after OGD/R and significantly inhibited by 25-HC. In addition, as shown in the Western blot analysis, 25-HC significantly inhibited cleaved caspase-3 segmentation in OGD/R-induced PC12 cells and elevated the Bcl-2/Bax ratio. Furthermore, the results of this study suggest that 25-HC may reverse OGD/R cytotoxicity.

**Figure 4:**
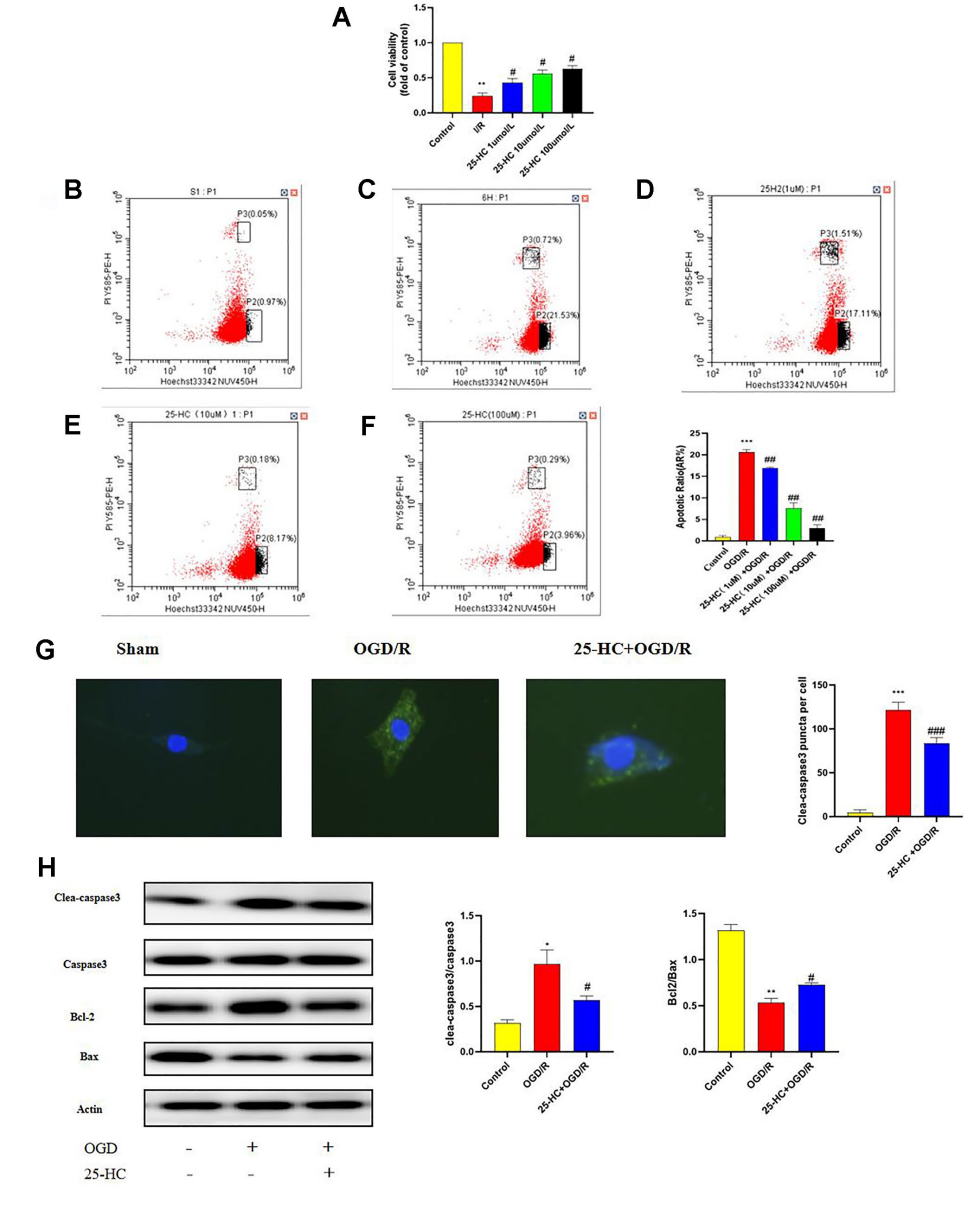
**25-Hydroxycholesterol (25-HC) protected PC12 cells from I/R-induced cytotoxicity and inhibits autophagy following oxygen-glucose deprivation/reperfusion (OGD/R) in PC12 Cells.** (**A**) PC12 cell viability is assessed by measuring CCK8. Results were shown as fold of control. (**B**–**F**) PC12 cells were pretreated with 25-HC and then cultured under OGD/R conditions or normoxia. Apoptosis analysis was performed, and representative flow cytometry images are shown. Quantifications of the percentage of apoptotic cells are shown (N = 3 per group, ***P<0.001, **P<0.01 versus sham group, ##*P*<0.01, #*P*<0.05 versus OGD/R group). (**G**) The expression of cleaved caspase3 was tested by immunocytofluorescent staining (N = 3 per group, ***P<0.001 versus sham group, ###*P*<0.001 versus OGD/R group). (**H**) The expressions of cleaved caspase-3, caspase-3, Bcl-2, and Bax were measured by Western blot (N = 3 per group, **P<0.01, *P<0.05 versus sham group, #*P*<0.05, versus OGD/R group).

**Figure 4:**
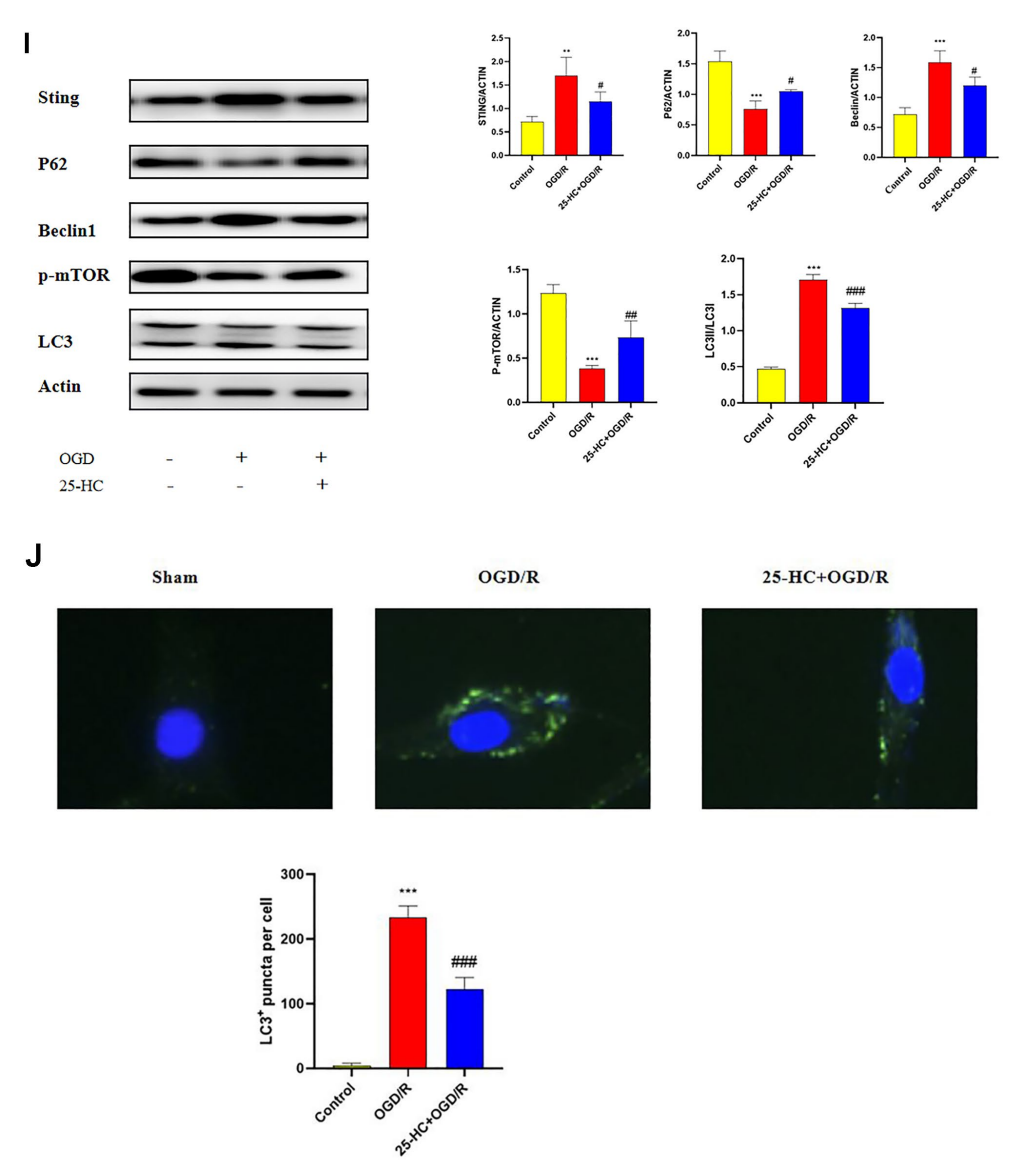
**25-Hydroxycholesterol (25-HC) protected PC12 cells from I/R-induced cytotoxicity and inhibits autophagy following oxygen-glucose deprivation/reperfusion (OGD/R) in PC12 Cells.** (**I**) The expression of p-mTOR, STING, P62, Beclin1, LC3 I, and LC3 II were tested by Western blot (N = 3 per group, ***P<0.001, **P<0.01 versus sham group, ##*P*<0.001, ###*P*<0.001, ##*P*<0.01, #*P*<0.05 versus OGD/R group). (**J**) The protein levels of LC3 were detected by immunofluorescence (N = 3 per group, ***P<0.001 versus sham group, ###*P*<0.001 versus OGD/R group).

### 25-HC inhibits autophagy following OGD/R in PC12 cells

Whether 25-HC modulates autophagy was verified in an *in vitro* ischemia model. To detect autophagy activity after OGD/R and determine whether 25-HC can inhibit autophagy activation, the protein expressions of LC3, Beclin1, and P62 in ischemic PC12 cells were examined. The expression levels of Beclin1 and LC3-II after OGD/R were significantly higher than those in the control group, whereas the expression levels of P62 and Bcl-2 were significantly lower than those in the control group. 25-HC (10 μM) significantly inhibited the expression of Beclin1 and LC3-II after OGD/R and enhanced the expression of P62 (Figure 4I). As indicated in Figure 4J, 25-HC (10 μM) pretreatment could effectively inhibit the increase in LC3 levels in PC12 cells induced by OGD/R.

### Involvement of the STING-mTOR/autophagy pathway in 25-HC-induced protection in PC12 cells

**Figure 5:**
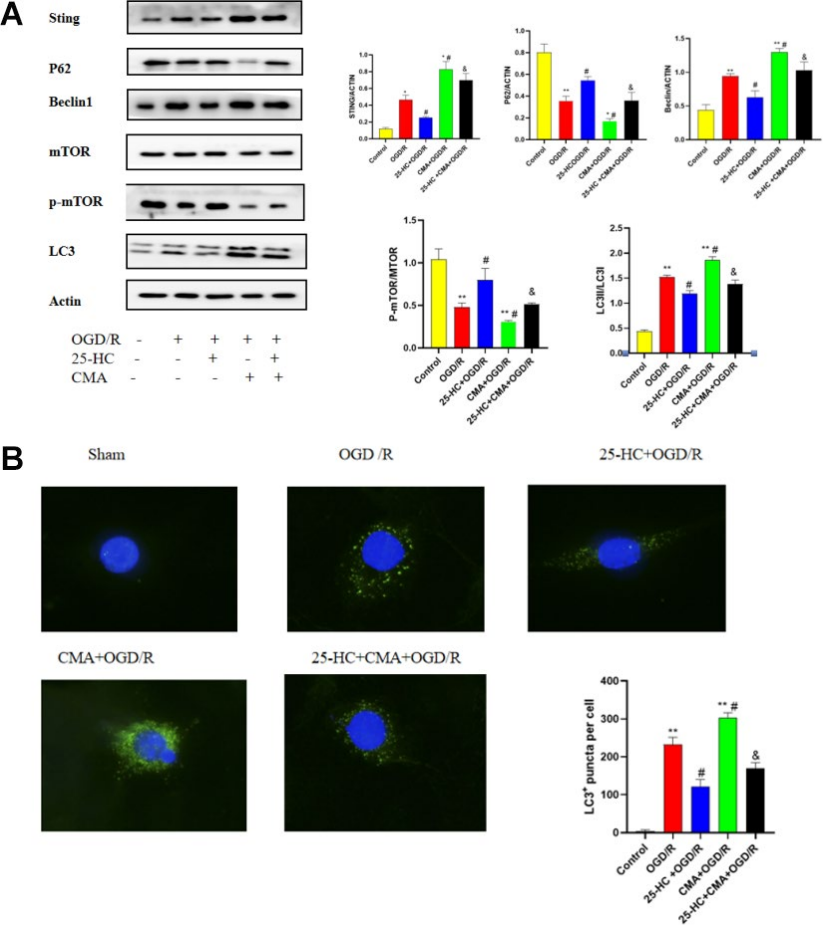
**Involvement of the STING-mTOR/autophagy pathway in 25-Hydroxycholesterol (25-HC)-induced protection in PC12 cells.** (**A**) The expressions of p-mTOR, STING, P62, Beclin1, LC3 I, and LC3 II were tested by Western blot (N = 3 per group, **P<0.01, *P<0.05 versus sham group, #*P*<0.05 versus OGD/R group, &*P*<0.05 versus OGD/R+CMA group). (**B**) The protein levels of LC3 were detected by immunofluorescence (N = 3 per group, **P<0.01 versus sham group, #*P*<0.05 versus OGD/R group, &*P*<0.05 versus OGD/R+CMA group).

We investigated whether 25-HC modulates autophagy by affecting the STING/mTOR pathway in PC12 cells. As shown in Figure 5A, 25-HC significantly reduced the OGD/R-induced increase in STING protein expression, and 25-HC significantly increased the molecule that decreased the OGD/R-mediated phosphorylation of mTOR. Notably, administration of the STING protein agonist CMA significantly inhibited the effect of 25-HC on OGD/R-induced p-mTOR expression. Subsequently, we investigated the role of the activation of the STING/mTOR pathway in 25-HC-mediated autophagy inhibition under OGD/R conditions. Data showed that the activation of the STING/mTOR pathway by CMA treatment significantly eliminated 25-HC-mediated autophagy inhibition, as demonstrated by the upregulated expressions of Beclin1, LC3-II, and STING protein P62 and the downregulation of p-mTOR. As indicated in Figure 5B, 25-HC (10 μM) pretreatment could effectively inhibit the increase in LC3 in PC12 cells induced by OGD/R. LC3 expression was increased after CMA administration. Taken together, these results suggest that STING/mTOR signaling is involved in 25-HC-mediated autophagy inhibition of OGD/R exposure in PC12 cells and subsequent neuroprotective effects.

## DISCUSSION

This study was carried out to determine the inhibitory effect of 25-HC on MCAO-induced neuronal apoptosis. The protective effects were mediated by autophagy through inhibition of the STING activity. To our knowledge, this study is the first to show that oxysterol can reduce cerebral ischemia-induced neuronal apoptosis and exert a cerebral protective effect. Ischemic stroke is generally known to increase death and disability. Zhang et al. [14] reported that cerebral ischemia is an important component of pathological damage attributed to ischemic stroke. Stroke accounts for 85% of all chronic adult disabilities and can lead to neuronal apoptosis [15, 16]. In addition, damage to the blood-brain barrier, neuronal damage, and vascular damage occur after cerebral ischemia reperfusion [17]. Thus far, effective treatments capable of reducing the adverse reactions associated with ischemic injury have been rarely proposed. Thus, using animal and cell culture models, the protective effect of 25-HC on cerebral ischemia injury was assessed by determining whether 25-HC is associated with inhibition of overactivated autophagy.

Brain injury is a common symptom of stroke [18]. The extract of *Gastrodia elata* reduces cerebral infarction and brain injury in rats with transient focal cerebral ischemia induced by MCAO [19]. According to Lu et al., 25-HC can significantly mitigate myocardial infarction caused by myocardial ischemia and reperfusion in mice [9]. Furthermore, pretreatment with vitexin reduces the infarction due to hypoxic-ischemic injury [20]. The present study showed that 25-HC significantly reduced the size of cerebral infarction in MCA-induced ischemic stroke mice.

Bax and Bcl-2 belong to the Bcl-2 family. Bcl-2 is an anti-apoptotic protein, and Bax acts as a pro-apoptotic protein. Caspases, a family of cysteine proteinases, are key mediators of nerve cell apoptosis and neurodegeneration [21]. Cleavage of caspase-3 is activated by I/R, leading to DNA fragmentation and mitochondrial dysfunction and thereby promoting cell death [22]. In the MCAO mouse model, olivine exerts anti-apoptotic effects by increasing the expression of Bcl-2 and decreasing the expression of Bax [23]. 25-HC can inhibit the upregulated expression of Bax and downregulated expression of Bcl-2 in I/R-induced myocardium [24], as well as downregulation of cleaved caspase-3/T-caspase-3 ratio. Similarly, our data showed that 25-HC inhibited the expression of cleaved caspase-3 and Bax and decreased the expression of Bcl-2 in MCAO mice. *In vitro* studies have shown that 25-HC can reduce the increase in apoptosis induced by I/R. Thus, 25-HC can protect against apoptosis in the mouse brain and PC12 cells.

Oxidative stress has been reported to cause brain injury [25]. In cerebral I/R models, the administration of ezetimibe mitigates MDA [26]. In an H_2_O_2_-induced oxidative damage model, vitexin can reduce MDA content [20]. MPO exists in PMN and is a specific enzyme in PMN. The amount of MPO contained in each PMN is relatively constant. Activation, migration, adhesion, and massive attachment of PMNs in vascular peripheral tissues are vital steps in the inflammatory response and exert critical effects during I/R injury [27]. Likewise, the results of this study showed that 25-HC reduced LDH, MDA, and MPO levels in mice with MCAO. These data suggest that 25-HC mitigates the oxidative damage caused by MCAO.

Autophagy is a catabolic process in which aging proteins and organelles in cells are destroyed via lysosome-dependent pathways to restore homeostasis [28]. Abnormal autophagy processes have been shown to induce numerous diseases [29]. Under normal physiological conditions, autophagy regulates intracellular homeostasis. However, in the presence of a disease, autophagy is activated, thereby playing a defensive and harmful role [30]. Autophagy is a double-edged sword with controversial functions in ischemic brain injury [31]. An increasing body of evidence suggests that increased autophagy is a harmful mechanism of I/R damage [32–34]. Accordingly, the regulation of autophagy may contribute to the prevention or treatment of ischemic stroke. As an important signaling molecule in immunity and inflammation, STING are important regulators of autophagy [35] performed *in vitro* experiments and found that virus-infected cells generate cyclic GMP-AMP-activated STING, STING ATG5 LC3 fat changes induced by activation of autophagy, and degradation of cytoplasmic DNA virus. The point mutations are activated to restrain the activity of STING, can reverse LC3 lipid, and inhibit autophagy attributed to cyclic guanosine monophosphate–adenosine monophosphate [36]. A study revealed that STING and autophagy activation are linked, and STING induced LC3 fat and activate autophagy. In this study, we found that 25-HC had an inhibitory effect on STING activity in *in vivo* and *in vitro* experiments. To our knowledge, this study is the first to report that 25-HC had a protective effect on brain nerve cell apoptosis induced by reperfusion injury through the inhibition of STING activity.

Excessive autophagy can lead to autophagic death, which can aggravate human diseases (e.g., atherosclerosis and stroke) [37]. In the presence of I/R injury, the beneficial effect of berberine is related to the inhibition of autophagy by reducing the expression of proteins associated with autophagy (i.e., Beclin1, BNIP3, and SIRT1) [38]. Gastrodin can significantly reduce the expression of autophagy markers LC3 and Beclin1 in astrocytes [39]. This study showed that 25-HC inhibits MCAO within the small mouse mTOR and the expression of p62, inhibiting expressions of STING, Beclin1, and Lc3 II. In addition, the effects of 25-HC on STING/mTOR pathway-related protein expression, oxidative damage, apoptosis, and inflammation can be reversed by CMA, suggesting that 25-HC inhibits autophagy through the STING/mTOR pathway, thereby alleviating MCAO-induced ischemic stroke.

## Conclusion

**Figure 6:**
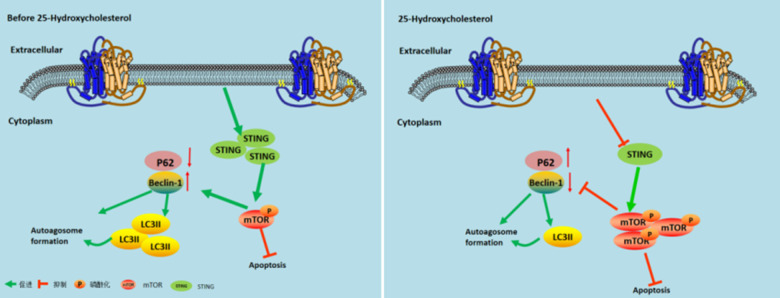
**Model describing the mechanism by which 25-Hydroxycholesterol protects against cerebral ischemiareperfusion injury.** Left Figure: After cerebral ischemia and reperfusion, the expression of STING protein is activated and mTOR phosphorylation is inhibited, thereby increasing the expression of autophagy and achieving cerebral ischemia effect. Right image: After administration of 25-hydroxycholesterol, the expression of STING protein was inhibited, and mTOR phosphorylation was activated, thus inhibiting the expression of autophagy and achieving the effect of cerebral ischemia tolerance.

This study showed that 25-HC alleviated cerebral infarction and apoptosis induced by MCAO. In addition, 25-HC can reduce the increase in LDH, MDA, and MPO levels induced by MCAO. It inhibited the inflammatory response in rats with MCAO by regulating secretion of proinflammatory cytokines (IL-1β, IL-6, and TNF-α). It can reverse the abnormal expression of proteins associated with autophagy (LC3, STING, p62, Beclin1, and p-mTOR). The results of this study suggest that 25-HC inhibits autophagy dysfunction via the mTOR/STING pathway, thereby alleviating MCAO-induced ischemic stroke (Figure 6). 25-HC may help develop new strategies for stroke prevention.

## MATERIALS AND METHODS

### Animals

The experiment scheme of the study was approved by the Ethics Committee of Animal Experiment of Wenzhou Medical University, in accordance with the Animal Experiment Guidelines of Wenzhou Medical University (No. WYDW2019-0539) C57BL/6 male mouse (20–25 g) was provided by Beijing Life River Experimental Animal Center. The experiment was conducted in a controlled environment (12-h light/dark cycle; 21.2° C). The animals were kept at 60%–70% humidity for 1 week and then received standard laboratory food and water free of charge.

### Models and drugs

Mice were anesthetized by intraperitoneal injection of sodium pentobarbital (50 mg/kg; Sigma, USA) with saline. The anesthetized mice were then ventilated with endotracheal intubation to assist breathing. Subsequently, a heating pad was used to maintain their rectal temperatures at 37° C. The internal carotid artery, external carotid artery, and right carotid artery were carefully separated after a median skin incision in the neck under an operating microscope. A 4-0 monofilament nylon wire coated with silicon was inserted into the right internal carotid artery and pushed forward to occlude the origin of the middle cerebral artery. A thread was used to hold the filament in place. When the operation procedure was completed, the neck wound was closed, and the animal was allowed to recover from anesthesia. Under isoflurane anesthesia, the vessels were pulled out for reperfusion for nearly 10 mm 1 h after ligation. Hemiplegia before reperfusion and temperature increase (37.8–38.8° C) were used to determine whether the plug was successful or not. Specific to sham-operated animals, an identical process was conducted with no embolization of the middle cerebral artery. The sham group did not undergo ligation, and the 25-HC group was subjected to I/R surgery+25-HC (soluble in normal saline; MCE, USA) by intraperitoneal injection. The control group received I/R surgery with a mixture of normal saline and ethanol of the same amount. In the oxygen-10-acridine acetic acid (CMA) group, CMA was injected 2 h after modeling, and the agonist stimulated the expression of STING protein at 250 μg/ml (I.P.). Sigma-Aldrich (St. Louis, MO, USA) provided the aforementioned drugs.

### Measurement of infarct size

After 24 h, reperfusion was conducted, excessive amounts of chloral hydrate were used for euthanasia, and the brain tissue was quickly removed to make 2 mm thickness after coronary section. Then, 2% 2,3,5-triphenyl four azole nitrogen (TTC) solution was used for dyeing 20 min at 37° C. Subsequently, 4% paraformaldehyde was used for the fixing process for 24 h, and imaging and scanning processes were conducted with the Image Pro Plus 6.0 software. Cerebral infarct volume was referred to as an infarct tissue percentage in overall brain tissue.

### Western blot

At 24 h after reperfusion, euthanasia was performed with chloral hydrate overdose, and the infarct-side hippocampus was rapidly separated. The operating process was conducted on a cold surface, and cell metabolism was stopped with liquid nitrogen. Tissue homogenates were treated with 1 PMSF (Beyotime, CHN), 10 phosphatase inhibitors (Roche, Germany), and 100 RIPA lysis buffer (Beyotime, CHN). The tissue extract underwent 30-min centrifugation under 12,000 g at 4° C. Western blot was conducted following the usual procedure. The protein was then transferred from the gel to a polyvinylidene fluoride membrane (Million, MA, USA) for the Western blot analysis. When closure was achieved, the sample was incubated by using primary antibody under 4° C throughout the night. The primary antibodies (Abcam, Cambridge, MA, USA) used were as follows: anti-cleaved caspase-3 (1:1000 dilution), anti-Bax (1:1000 dilution), anti-mTOR (1:2000 dilution), anti-p-mTOR (1:2000 dilution), anti-STING (1:1000 dilution), anti-p62 (1:1000 dilution), and anti-Beclin1 (1:1000 dilution). After the incubating process using a suitable horseradish peroxidase-conjugated secondary antibody, the antigen-antibody reacting process was detected using Quantity One analysis software (Bio-Rad, San Francisco, CA, USA) and an improved chemiluminescence system.

### Enzyme-linked immunosorbent assay (ELISA)

To perform ELISA, blood was obtained at the end of the experiment. A 10-min blood centrifugation was conducted at 3000 rpm for the collection of serum. Serum levels of tumor necrosis factor alpha (TNF-α), interleukin (IL)-6, and IL-10 were obtained using ELISA kits (R&D Systems, Minneapolis, USA), which were obtained commercially by complying with manufacturer’s guidelines.

Determination of myeloperoxidase (MPO), malondialdehyde (MDA), and lactate dehydrogenase (LDH) levels MPO, MDA, and LDH levels in the serum were quantified by using commercial kits following the instructions of the manufacturer.

### Cell culture and induction of oxygen–glucose deprivation

Neuron-like rat pheochromocytoma cell line PC12 cells (Manassas, VA, USA) in DMEM supplemented with 10% fetal bovine serum, antibiotics (penicillin, 100 IU/mL), and streptomycin (100 μg/mL) were cultured at 37° C in 5% CO_2_. PC12 cells (density of 1×10^4^cells/ml) were grown in DMEM containing 25-HC or inhibitors under constant oxygen incubation for 6 h when reperfusion was achieved, without serum glucose DMEM culture instead of neurons in the Petri dish culture standard. Cells were then transferred to an aerobic chamber (95% CO_2_ and 5% N_2_) for 6 h at 37° C. During reperfusion, the cells were placed in a normoxic incubator (95% air and 5% CO_2_), and normal neuron cultivation was then used to carry out the standard cultures for 24 h. The number of total cells in the cell suspension was determined using a disposable hemocytometer (Thermo Fisher Scientifific).

### Investigation for cell viability

Cells in 96-well plates (5000 cells/well) were treated under oxygen-glucose deprivation/reperfusion (OGD/R) conditions alone or in combination with 25-HC. CCK-8 kit (ZETE Life Inc., Menlo Park, CA, USA) was utilized for cell viability assessment. Optical density values were read at 450 nm by a microplate reader (Bio-Rad, Hercules, CA, USA).

### Determination of apoptosis

To examine the levels of apoptosis, the culturing and treating processes for PC12 cells were performed by using curcumin under various concentrations (100 and 200 mg/kg). Then, 72 h later, cells were washed using phosphate-buffered saline (PBS) and stained based with Annexin V-FITC/PI (ab14085, Abcam; 330 Cambridge Science Park Rd, Milton, Cambridge CB4 0FL, UK). Moreover, based on flow cytometry, this study measured the percentages of apoptotic and necrotic cells.

### Immunocytochemistry

In cultured neurons of PC12 cells, immunofluorescence staining was performed. In brief, neurons were subjected to the 15-min fixing process using PBS supplemented by 4% paraformaldehyde, and the 10-min permeabilizing process was performed using 0.2% Triton-100. PC12 cells were subjected to the blocking process with 5% bovine serum albumin (BSA) in PBS for 1 h at ambient temperature and subsequently incubated throughout the night using primary antibody inside PBS supplemented by 1% BSA. After three washes with PBS, a fluorescent secondary antibody was added for 1 h. Moreover, after the three washes with PBS, the plate was sealed by using DAPI. Fluorescence images were captured under an OLYMPUS BX51 fluorescence microscope. Gain, threshold, and black levels did not change during the respective experiments. All image investigations were carried out based on the experiment conditions.

### Statistical analysis

GraphPad Prism (version 8.01, GraphPad Software Inc., La Jolla, CA, USA) was used in the data presentation and statistical investigation. However, the sample size was not determined. Data are expressed as the mean ± standard error of the mean. Based on Student’s t-test and analysis of variance, diversifications of two or more groups were separately analyzed. Statistical significance was set at *P*<0.05.

### Data availability statement

The raw data supporting the conclusions of this manuscript will be made available by the authors, without undue reservation, to any qualified researcher.

### Ethics statement

All experimental protocols were performed according to the National Institutes of Health Guide for the Care and Use of Laboratory Animals, and all methods were approved by the ethics committee of the Laboratory Animal Ethics Committee of Wenzhou Medical University.
